# A Novel Treatment Protects *Chlorella* at Commercial Scale from the Predatory Bacterium *Vampirovibrio chlorellavorus*

**DOI:** 10.3389/fmicb.2016.00848

**Published:** 2016-06-20

**Authors:** Eneko Ganuza, Charles E. Sellers, Braden W. Bennett, Eric M. Lyons, Laura T. Carney

**Affiliations:** ^1^Microbiology Group, Heliae DevelopmentLLC, Gilbert, AZ, USA; ^2^Molecular Ecology Group, Heliae DevelopmentLLC, Gilbert, AZ, USA

**Keywords:** algae industry, *Chlorella*, crop protection, *Micractinium inermum*, pH-shock, predatory bacterium, *Vampirovibrio chlorellavorus*

## Abstract

The predatory bacterium, *Vampirovibrio chlorellavorus*, can destroy a *Chlorella* culture in just a few days, rendering an otherwise robust algal crop into a discolored suspension of empty cell walls. *Chlorella* is used as a benchmark for open pond cultivation due to its fast growth. In nature, *V. chlorellavorus* plays an ecological role by controlling this widespread terrestrial and freshwater microalga, but it can have a devastating effect when it attacks large commercial ponds. We discovered that *V. chlorellavorus* was associated with the collapse of four pilot commercial-scale (130,000 L volume) open-pond reactors. Routine microscopy revealed the distinctive pattern of *V. chlorellavorus* attachment to the algal cells, followed by algal cell clumping, culture discoloration and ultimately, growth decline. The “crash” of the algal culture coincided with increasing proportions of 16s rRNA sequencing reads assigned to *V. chlorellavorus*. We designed a qPCR assay to predict an impending culture crash and developed a novel treatment to control the bacterium. We found that (1) *Chlorella* growth was not affected by a 15 min exposure to pH 3.5 in the presence of 0.5 g/L acetate, when titrated with hydrochloric acid and (2) this treatment had a bactericidal effect on the culture (2-log decrease in aerobic counts). Therefore, when qPCR results indicated a rise in *V. chlorellavorus* amplicons, we found that the pH-shock treatment prevented the culture crash and doubled the productive longevity of the culture. Furthermore, the treatment could be repeatedly applied to the same culture, at the beginning of at least two sequential batch cycles. In this case, the treatment was applied preventively, further increasing the longevity of the open pond culture. In summary, the treatment reversed the infection of *V. chlorellavorus* as confirmed by observations of bacterial attachment to *Chlorella* cells and by detection of *V. chlorellavorus* by 16s rRNA sequencing and qPCR assay. The pH-shock treatment is highly selective against prokaryotes, and it is a cost-effective treatment that can be used throughout the scale up and production process. To our knowledge, the treatment described here is the first effective control of *V. chlorellavorus* and will be an important tool for the microalgal industry and biofuel research.

## Introduction

*Vampirovibrio chlorellavorus* is a non-photosynthetic cyano bacterium (*Melainabacteria*; Soo et al., 2014) with a predatory lifestyle that targets a variety of *Chlorella* species (Coder and Starr, 1978). This predator only feeds on living algae cells and is unable to grow in liquid or agar media unless co-cultured with the alga. Electron microscopy-based studies (Coder and Goff, 1986; Mamkaeva and Rybal’chenko, 1979) have shown that *V. chlorellavorus* uses a flagellum to reach its prey and attach to the surface of the alga through fibril appendages. The attached cells remain epibiotic contrary to other predatory bacteria belonging to *Bdellovibrio* or *Daptobacter* (Guerrero et al., 1986). The unique predation mechanisms of *V. chlorellavorous* are exquisitely regulated. The bacterial cell secretes a path that connects with the alga, breaches through the parent cell wall and if necessary also through the daughter cell wall (Coder and Goff, 1986). Recent metagenomic analyses suggest that plasmid DNA and hydrolytic enzymes are transferred to the prey cells via the T4SS secretion system where they apparently degrade the algal cell contents (Soo et al., 2015). As the *Chlorella* cell is digested, the color changes from dark green to yellow brown, and empty or “ghost" *Chlorella* cells accumulate in the culture. If left unabated, the majority of *Chlorella* cells are destroyed and the culture develops a granular texture due to progressive clumping of algal cells visible to the naked eye (Coder and Starr, 1978).

Early detection by optical microscopy is difficult due to the pleomorphic shape and the small size of *V. chlorellavorus* (<1 μm^3^; ultramicrobacterium; Coder and Starr, 1978). Other bacterial epiphytes may be mistaken for *V. chlorellavorus*; however, the infection becomes more apparent with the rapid increase in the proportion of *Chlorella* cells with attached bacteria and the development of a clear zone that spreads through the infected cell from the site of attachment (Coder and Goff, 1986). Using electron microscopy, *V. chlorellavorus* was first described in freshwater samples from a reservoir in Ukraine (Gromov and Mamkaeva, 1972) and later in freshwater anuran amphibian ponds in Brighton, UK (Wong et al., 1994). However, traditional detection tools have failed to provide a definitive diagnosis, and it is likely that the incidence of *V. chlorellavorous* infection has been overlooked. For example, only a few original papers have been published since this predator was first described (Gromov and Mamkaeva, 1972). Recently, molecular tools have targeted this predator across diverse systems. For example, *V. chlorellavorous* was identified in soil samples from Anhui, China (Shi et al., 2015), in bovine rumen samples from Nagaland, India (Das et al., 2014) and from open pond cultures of *Chlorella* at three different universities in the southwestern USA which suffered repeated *V. chlorellavorus* infections (personal communication from Drs. P. Lammers, J. Brown, and M. Sommerfeld).

In recent years it has become apparent that algal crop protection is one of the most important challenges facing the algal industry (Carney and Lane, 2014; McBride et al., 2016). *Chlorella* was the first eukaryotic microalgae to be grown in pure culture (Beijerinck, 1890) and today it is arguably the most common alga grown by the microalgal industry. Its high productivity and robustness allows a wide range of applications including food, feed, fertilizer, wastewater remediation, CO_2_ capture, and biodiesel production (Safi et al., 2014). *V. chlorellavorus* attacks this alga whether it is growing photoautotrophically (Coder and Goff, 1986), heterotrophically (Wong et al., 1994), or as described here, mixotrophically. Therefore, the lack of treatment to control *V. chlorellavorus* is a major challenge for the industry. Here, we report the development of tools for early detection and treatment of *V. chlorellavorous*.

## Materials and Methods

### *Chlorella* Culture Conditions

#### Identification of the Algae

*Chlorella* HS26 was isolated from soil samples in the Sonoran Desert of Arizona, USA and privately deposited in the Culture Collection of Algae at the University of Cologne (Germany). Total genomic DNA was extracted and purified using the DNeasy Plant Mini Kit (Qiagen, Hilden Germany) and the nuclear-encoded rRNA-operon was amplified by polymerase chain reaction (PCR) using DreamTaq^TM^ DNA Polymerase (Fermentas, St. Leon-Rot, Germany). The DNA was sequenced for 18S, ITS1, 5.8S, ITS2, 28S regions using universal eukaryotic primers (see Marin et al., 2003; Marin, 2012). The following PCR protocol was used: an initial denaturation step (95°C for 180 min) was followed by 30 cycles including denaturation (95°C for 45 min), annealing (55°C for 60 min), and elongation (72°C for 180 min). PCR products which showed single clear bands by gel electrophoresis were purified using the Dynabeads M-280 Streptavidin system (Holmberg et al., 2005). For sequencing the SequiTherm EXCEL II Long Read DNA Sequencing Kit (Biozym Diagnostik, Germany) and fluorochrome-labeled primer combinations were used. Two partial and overlapping sequences of each strand were read out with a LI-COR IR2 DNA Sequencer (LI-COR Biosciences, Lincoln, NE, USA) and assembled to the complete rDNA sequence using the program AlignIR^TM^ 2.0 (LI-COR Biosciences, Lincoln, NE, USA). Sequences were aligned manually on the basis of conserved rRNA secondary structures using SeaView 4.3.0 software and only unambiguously aligned sequences were used for phylogenetic analysis. The sequences were compared with existing sequences on the NCBI GenBank database via BLAST. The closest BLAST hits had at least 99 % similarity to *Chlorella* sp. NIES2171 (accession number AB731604) and *Chlorella vulgaris* CCAP/79 (FR865683), consequently the species was preliminary identified as *Chlorella* sp. HS26. It should be noted that recently, the two reference species were proposed as *Micractinium inermum* NIES2171 (Hoshina and Fujiwara, 2013) and *Micractinium* sp. CCAP 211/79 (Germond et al., 2013), respectively. The new sequences for the strain used in this work were deposited in the NCBI database under the accession number KU641127.

#### Laboratory Culture Conditions

Small-scale testing of *Chlorella* was performed in duplicate using baﬄed experimental Erlenmeyer flasks (100 ml volume), incubated at 25°C, and shaken at 100 rpm. The cultures were inoculated at 10% v/v from an axenic 2–3-day-old exponentially growing autotrophic flask culture using BG-11 medium. The experimental flasks were illuminated with LED lights at an intensity of 100 μmol photons/m/s, and sodium acetate trihydrate (2.5 g/L) was added daily to support mixotrophic conditions. The pH was maintained between 7 and 8, and the flasks were maintained in a CO_2_ enriched (2 %) atmosphere inside a chamber. For each flask experiment, we tested for a treatment effect, a time effect, and a treatment by time interactions using linear mixed models. Treatment and time were modeled as fixed effects, and we included a first-order autoregressive error structure (AR1) to account for temporally autocorrelated measures. Models were fit using the package lme4 (Bates et al., 2015) in R version 3.0.2 (R Core Team, 2016).

#### Open Pond Culture Conditions

The pH treatment validation was performed outdoors in open pond raceways (1000 L running volume) measuring 3.5 m long, 1.75 m wide and 19 ± 1 cm depth cultures. The mixotrophic cultures were fed acetic acid on demand through a pH-auxostat feedback control system. The culture was maintained at pH 7.4 ± 0.05 and residual acetic acid (0.2–1 g/L) and nitrate (0.2–0.5 g/L) were maintained at constant concentrations throughout the batch cycle using a feedstock solution of acetic acid (40% v/v) and NaNO_3_ (40 g/L) as titrants. The initial BG-11 medium was modified to contain (in g/L): sodium acetate (0.5), NaNO3 (0.5), K_2_HPO_4_ 3H_2_O (0.04), MgSO_4_ 7H_2_O (0.075), CaCl 2H_2_O (0.036), citric acid (0.006), ferric ammonium citrate (0.006), MgNa_2_EDTA H_2_O (0.001), and 1 ml/L of a trace metal solution. The trace metal solution contained (in g/L): H_3_BO_3_ (2.86), MnCl_2_ 4H_2_O (1.81), ZnSO_4_ 7H_2_O (0.22), CuSO_4_ 5H_2_O (0.079), Na_2_MoO_4_ 2H_2_O (0.391), Co(NO_3_)_2_ 6H_2_O (0.0494). The medium was prepared using reverse osmosis water and initial pH was corrected to 7.5 using HCl (1 M). The raceways were inoculated with 10% v/v of outdoor open cultures that had previously been exposed to *Vampirovibrio chlorellavorus*. Temperature was maintained at 24 ± 2°C using a 6 m long stainless steel heat exchange coil. Mixing was applied with a paddlewheel at 1 m/s tip speed. The cultures were aerated (0.05 vol air/vol culture per min) using a 6 m long porous hose and evaporation was corrected daily using reverse osmosis water. Culture conditions above were scaled up to a pilot (60,000 L) and commercial reactor (130,000 L) according to (Tonkovich et al., 2014, International Patent No 2014/144270 A1).

### Growth Assessment

#### Cell Dry Weight

Cell dry weight samples (10 ml) were collected in duplicates daily from the cultures and vacuum filtered with glass microfiber filter papers designed to retain particles of 1.1 μm (Ahlstrom^TM^ Grade 161). The filtrate was washed twice with 10 ml of ammonium bicarbonate 0.5 M solution and placed in an oven (105°C) until weight was stable.

#### Residual Nutrients

Culture samples (2 mL) were centrifuged (17,000 ×*g* for 7 min) and the supernatant was removed and diluted 20-fold. Acetate in the medium was analyzed by HPLC according to the Association of Official Agricultural Chemists Official Method 986.13. The nitrates were analyzed using a Latchat Quickchem^TM^ 8500 and the UV-method 10049 (Hach, Milwaukee, WI, USA).

#### Culture Longevity

Culture longevity of *Chlorella* was determined based on the total productive days in the target reactor. *Chlorella* cultures operated in sequential batch cycles. The new batch started from the previous batch before this reached stationary phase. Thus, those days with stable or declining dry weights indicated the end of the life of that culture and were excluded from the longevity calculations. The longevity within our commercial reactors (130,000 L) was compared between treated (*n* = 9) and untreated (*n* = 8) runs using a student’s *t*-test (JMP^®^, Version 12.1 SAS Institute Inc., Cary, NC, USA).

### Contamination Assessment

#### Total Aerobic Bacterial Counts

Total aerobic bacterial counts were determined in duplicates using 3M Petrifilm^TM^ Aerobic Count Plates. The plates were incubated for 3 days at 35°C and counts were read using the 3M^TM^ Petrifilm^TM^ Plate Reader and associated image analysis software.

#### The Percentage of *Chlorella* with Attached Bacteria

The percentage of *Chlorella* with attached bacteria was determined with phase contrast light microscopy using oil immersion objective lens (100×; Olympus DP72). Algal cells with one or more bacteria on their surface were recorded as positive infection. Overall, 50–100 algal cells were used to calculate the percentage. The standard deviation for the method was below 10%.

#### Determination of Bacterial Community Structure

Samples (2 mL) were collected daily from outdoor *Chlorella* cultures and prepared for small subunit rDNA sequencing as follows. For total DNA extraction, the biomass was concentrated by centrifugation (10,000 ×*g* for 10 min) and DNA was purified using the ZR Fungal/Bacterial DNA mini prep kit (Zymo Research, Irvine, CA, USA). To isolate microbiome DNA that was only associated with the phycosphere, biomass was concentrated using slow speed centrifugation (1000 ×*g* for 7 min), the supernatant containing unattached bacteria was discarded, the pellet was washed by resuspension in sterile water three times, and the final pellet was collected using high speed centrifugation (17,000 ×*g* for 7 min). To isolate DNA from the whole culture portion, biomass was collected using high speed centrifugation (17,000 ×*g* for 7 min). For all samples, DNA purity and quantity was determined via 260/280 nm readings then normalized to 10 ng/μL. PCR was conducted on the normalized gDNA using chloroplast excluding 16S rDNA primer set 799F/U1492R (Chelius and Triplett, 2001). The following PCR protocol was used: an initial denaturation step (98°C for 30 s), followed by 35 cycles of denaturation (98°C for 10 s), annealing (53°C for 30 s) and elongation (72°C for 60 s). The PCR cycle was ended with a 72°C final elongation for 10 min. The resulting PCR product was visualized on agarose gels for confirmation of expected bands (∼700 bp) and then cloned using Zero Blunt Topo PCR cloning kits (Invitrogen, Carlsbad, CA, USA). 16S rDNA clone library inserts were sequenced with the T7 vector primer using an ABI 3730xl DNA Analyzer.

The resulting 16S rDNA sequences were aligned and trimmed of primer and vector sequences using the built-in trimming tool in the Geneious 8.1.5 software suite (Biomatters, Ltd, New Zealand) with vector trimming against the NCBI UniVec database ^1^ Sequences with an HQ % quality score below 30% were excluded from analysis. Sequences were then aligned to the NCBI “nr” database for taxonomic assignment using BLAST (Altschul et al., 1990). Best BLAST hits had ≥98% similarity to the query sequence picked for taxonomic assignment. For verification of assignments, the trimmed sequences were also aligned and classified using the SINA online alignment tool against the SILVA 119 database [lc1] (Pruesse et al., 2012). Although these methods involved two different reference databases and alignment algorithms, the taxonomic assignments were in agreement at >98–99% sequence similarity (data not shown). Heatmaps of bacterial relative abundance were generated using the phyloseq R package (McMurdie and Holmes, 2013).

#### Predator Identification and Tracking Using qPCR Assay

A proprietary 6-carboxyfluorescein based (FAM) qPCR assay was designed for *V. chlorellavorous* consisting of forward and reverse primers and a species specific probe. The assay is not publically available at this time; however, the information may be made available by contacting Heliae and agreeing to confidentiality terms. The assay was designed using a clone insert bacterial 16S rRNA sequence from an infected pond (Accession number KU570459). The clone exhibited >99% similarity (692/695 nucleotide match) to a GenBank 16S gene sequence (Accession number HM038000) from the *V. chlorellavorous* type culture that was deposited by Coder and Starr (1978) and sequenced by the American Type Culture Collection (ATCC29753). DNA collected from the phycosphere-enriched fraction of the same culture was used as a positive control template. Pond samples (2 mL) were lysed by bead beating using 0.5 mm beads at 3400 rpm for 2 min and centrifuged at 10000 ×*g* for 1 min to remove cellular debris. The aqueous supernatant (1 μL) from the lysate was used as a template for the qPCR assay in 10 uL total volume reactions. The following qPCR protocol was used: an initial denaturation step (98°C for 3 min), followed by 40 cycles of denaturation at 95°C for 5 s, and annealing at 60°C for 30 s. The total run time for the assay was 120 min. The assay showed specificity only to the *V. chlorellavorous* assigned clone insert and tested negative (*C*_t_ = 0) against purified 16S sequences from various bacterial isolates representing multiple genera (*Shewanella* sp., *Acinetobacter* sp., *Ochrobactrum* sp., *Pseudochrobactrum* sp., *Bacillus* sp., *Stenotrophomonas* sp., *Clostridium* sp., *Azospirillum* sp., *Gemmobacter* sp., *Pseudomonas* sp., *Pedobacter* sp., *Pannonibacter* sp., *Rheinheimera* sp. *Azoarcus* sp., *Cloacibacterium* sp., and *Comamonas* sp.).

### Predator Treatment Development

#### Literature Review on Cytoplasmic pH Regulation by Microalgae

Literature review on cytoplasmic pH regulation by microalgae was performed to compare the response of Cyanophyta and Chlorophyta to the medium pH. The review included peer-reviewed articles that analyzed the cytosolic pH of algae at two or more medium pH set points. Cytosolic pH was analyzed using either 5,5-dimethoxazolidine-2,4-dione (DMO), ^31^P-NMR spectroscopy, or fluorochrome methods. Mean pH values were easily transcribed either from a data table or a detailed line graph reported in those articles. Means and standard deviations were calculated based on data from 3 to 7 different strains. Acidophilic microalgae, with an optimum growth at a pH below 5, were excluded from this review. Most cyanobacteria stopped growing at medium pH below 6 and cytosolic pH data could not be retrieved for those data points.

#### The pH-Shock Treatment

The pH-shock treatment was applied by adding hydrochloric acid (HCl; 34% v/v) to well mixed algae culture until the pH decreased from 7.5 to 3.5. The pH of the algae culture was maintained at pH 3.5 for 15 min in the presence of 0.5 g/L residual acetate. The pH was then returned to pH 7.5 using sodium hydroxide (2 M). More details on the method and method variants are provided by Ganuza and Tonkovich (2015, U.S. Patent No 9,181,523).

## Results

### Predator Identification

**FIGURE 1:**
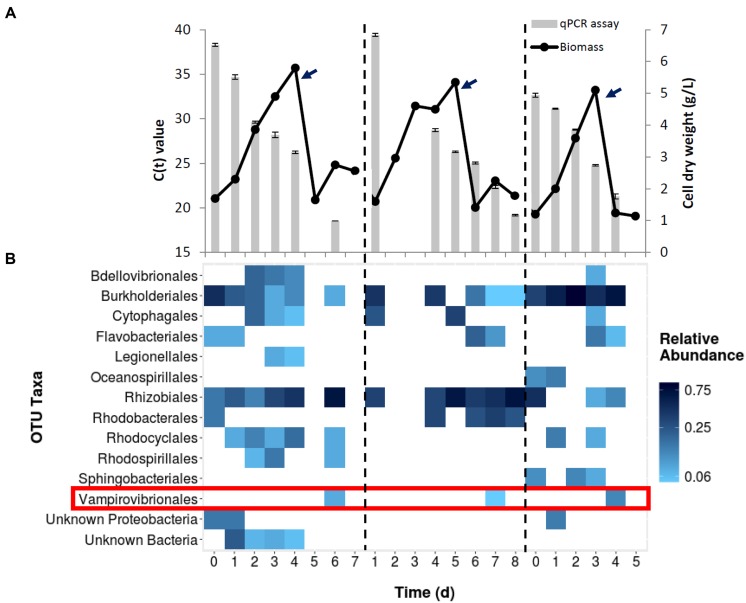
**(A)** Growth of *Chlorella* culture during three different scale up cycles in outdoor mixotrophic commercial reactors. Each cycle (divided by the dash line) represents the seed production (60,000 L) and transfer (denoted by arrows) to a commercial-scale reactor (130,000 L volume). Bars represent *Vampirovibrio chlorellavorus* infection as detected by qPCR assay in each cycle. The *C*_t_ value, or cycle threshold, decreases as the target abundance increases. **(B)** Bacterial community structure from whole culture samples of same three runs based on 16s rRNA gene sequencing. The detection of *V. chlorellavorus* sequences coincide with a decline or arrest in the growth of *Chlorella* shown in **(A)**.

Three data sets are presented as representative examples (**Figure 1**). Cultures grown in 60,000 L bioreactors showed initial *Chlorella* growth as measured by biomass increase. After transferring the cultures to the 130,000 L reactor (arrows; **Figure 1A**), biomass declined in 2–3 days. When identifying *V. chlorellavorus* using 16S rRNA sequences, the bacterium was not detected until day 6, at which time the culture was crashing. In addition to *V. chlorellavorus*, other bacterial taxa were present throughout the experiments (**Figure 1B**). When quantifying *V. chlorellavorus* using the qPCR assay the progress of the infection was observed in anticipation of the crashing of the culture (**Figure 1A**).

**FIGURE 2:**
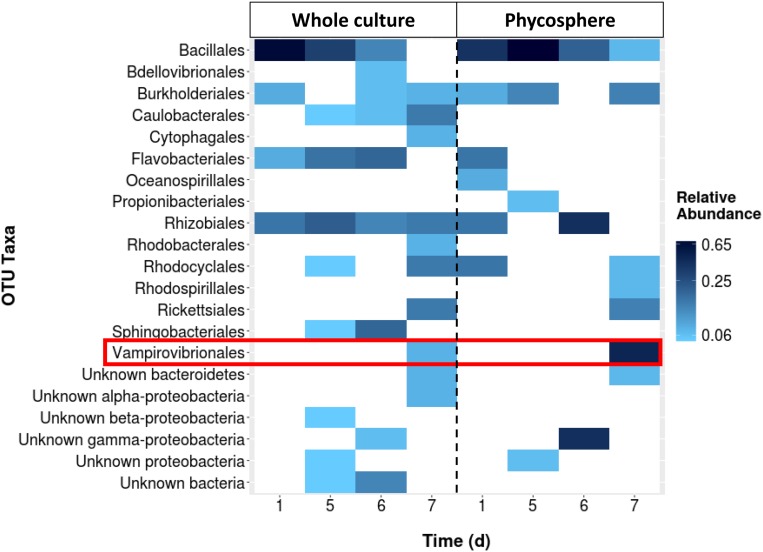
**The comparison between the whole culture bacterial community structure (16s rRNA gene sequencing assay) of a batch culture of *Chlorella* and the corresponding phycosphere portion.** Sequencing data was not available for days 2–4.

A fourth 130,000 L reactor was investigated using only 16S rRNA sequences and the proportion of *V. chlorellavorus* from the whole pond community was compared to the community associated with the phycosphere (**Figure 2**). While *V. chlorellavorus* was present in both whole pond and phycosphere samples on day 7, the proportion of reads assigned to this predatory bacterium was much more abundant in the phycosphere sample (0.47 versus 0.07).

**FIGURE 3:**
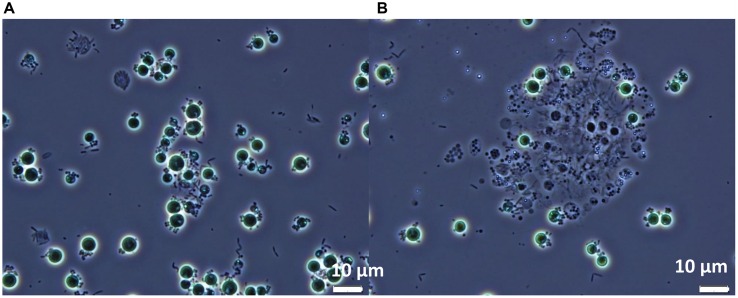
**Phase contrast micrographs of *Chlorella* commercial cultures heavily infested with *Vampirovibrio*-like cells at two different stages (A) Appearance of the first empty or ghost cells. (B)** Culture clumping. Micrographs produced by R. A. Andersen.

When viewed microscopically, the *Chlorella* culture was heavily infested with *Vampirovibrio*-like cells (**Figure 3**). Initially, a single *V. chlorellavorus* cell attached to the outside of a *Chlorella* cell, but this quickly developed into a chain and then a cluster of bacterial cells. As the predation proceeded, numerous bacterial cells formed and then were released, eventually leaving empty or ghost algal cells (**Figure 3A**). Finally, as the predation event neared completion, the *Chlorella* cells began clumping, apparently held together by a bacterial matrix (**Figure 3B**).

#### Predator Treatment Development in Laboratory

**FIGURE 4:**
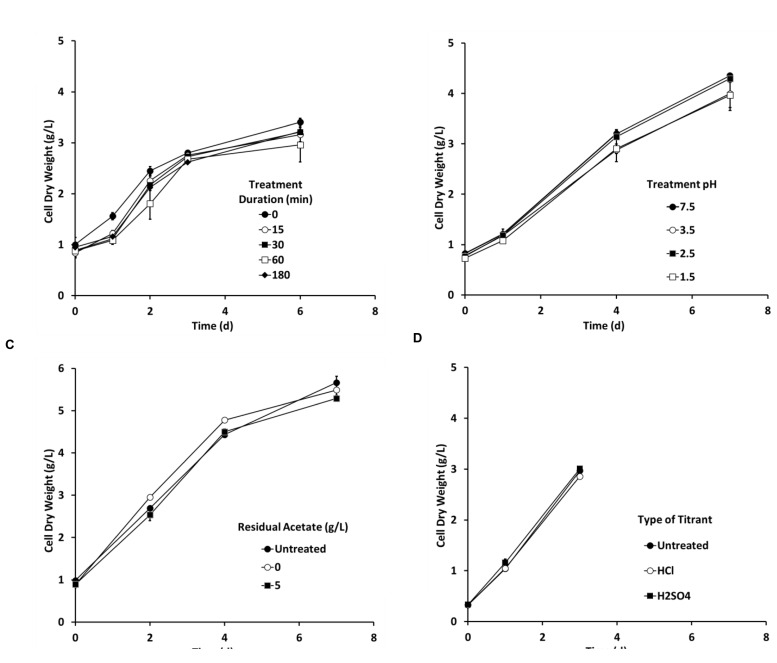
**The pH-shock treatment (A) duration, (B) pH level, (C) residual acetate concentration, and (D) type of titrant did not affect *Chlorella* growth in flasks (250 mL, *n* = 2).** Unless stated otherwise the pH treatment was conducted using hydrochloric acid (HCL) to reduce pH to 3.5 for 15 min in the presence of 0.5 g/L acetate and then inoculated aseptically into the *Chlorella* culture. Error bars represent 1 standard deviation.

Preliminary flask experiments showed that *Chlorella* tolerated a pH-shock (pH 3.5) up to at least 2 h (**Figure 4A**). *Chlorella* also tolerated a 15 min pH-shock as low as pH 1.5 (**Figure 4B**), and cells tolerated the pH-shock even in the presence of up to 5 g/L acetate (**Figure 4C**). Lastly, *Chlorella* tolerated treatments using either sulfuric acid or HCls as titrants (**Figure 4D**). No significant differences were observed between any of the treatments tested during these four experiments according to the Generalized Linear Model analysis (*P* > 0.1).

#### Predator Treatment Development in Open Ponds

**FIGURE 5:**
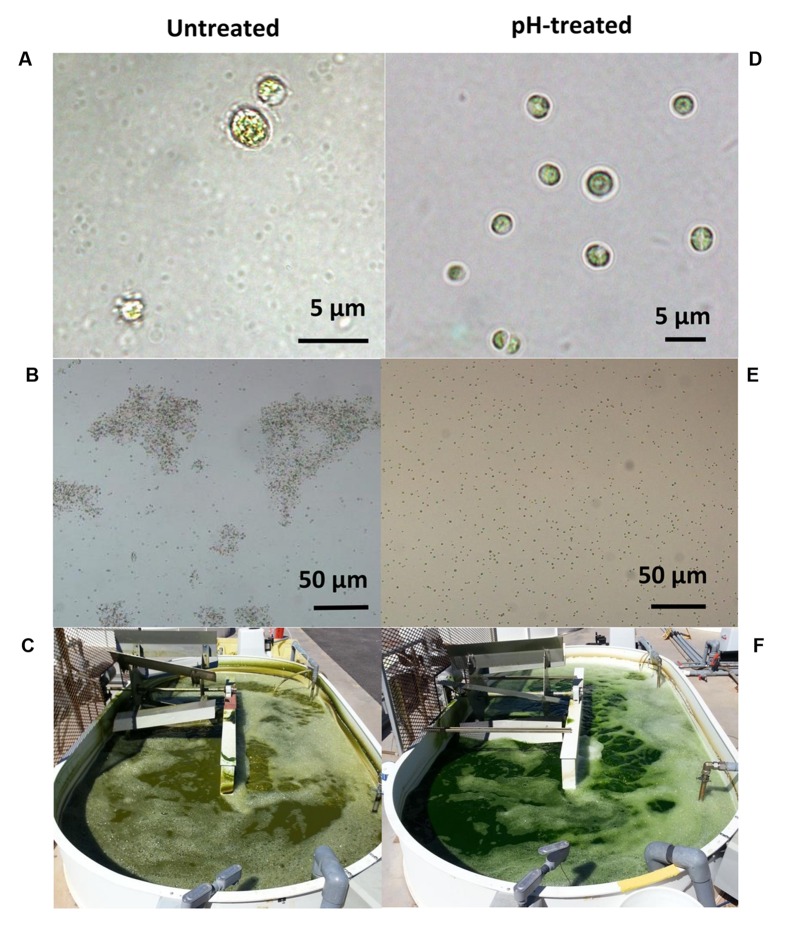
**The symptoms associated with *Vampirovibrio chlorellavorus* infection such as (A) bacterial attachment to the algae (B), clumping of algae cells and (C) change in the coloration of the culture (C) were reverted by the pH-shock treatment (D–F, respectively).** Pictures and micrographs correspond to a simultaneous side by side comparison of raceways (1000 L, **C,F**) containing untreated (left) or pH-treated (right) culture derived from an industrial scale reactor (130,000 L). Micrograph 5A was produced by J. Wilkenfeld.

**FIGURE 6:**
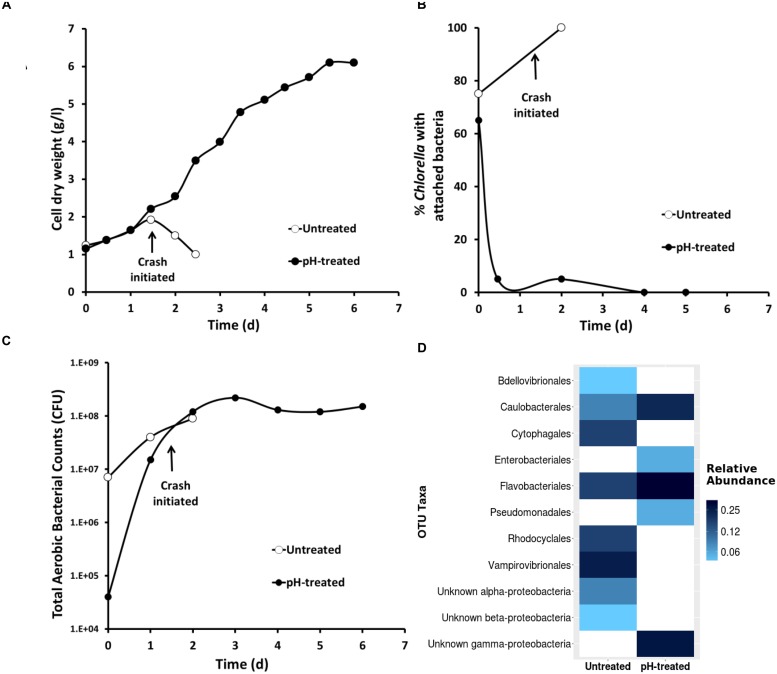
**The impact of pH-shock treatment (close circles) on (A) *Chlorella* growth (B) bacterial attachment to the algae phycosphere (C) total aerobic bacterial count and (D) 16s rRNA *Vampirovibrio chlorellavorus* sequencing.** Data correspond to a simultaneous side by side comparison in raceways (1000 L, *n* = 1) of pH-treated and untreated culture originated from in an industrial scale reactor (130,000 L).

Based on positive results from laboratory experiments, the pH-shock treatment was tested outdoors on mixotrophic cultures growing in small raceways (1000 L). A contaminated culture (70% *Chlorella* cells having attached bacteria) was removed from a large reactor (130,000 L) and used to inoculate two small raceways. One raceway was pH-treated as follows: (1) the pH was decreased from 7.5 to 3.5 using HCl, (2) the pH was maintained at pH 3.5 for 15 min in the presence of 0.5 g L^–1^ residual acetate, and (3) the pH was then returned to pH 7.5 using sodium hydroxide (Ganuza and Tonkovich, 2015, U.S. Patent No 9,181,523). The second raceway was left untreated. The visual symptoms associated with *V. chlorellavorus* predation (i.e., bacterial attachment to the algae, clumping of algae cells, and change in culture color) were only observed in the untreated raceway (**Figures 5A–C**), and this culture crashed within 2 days. In the pH treated culture, the symptoms of infection were reversed and a culture crash was prevented (**Figures 5D–F**). The pH-treated culture remained healthy, reaching a cell density of 6 g/L within 6 days (**Figure 6A**). Bacterial attachment to the algal cells was reversed and eliminated within 12 h of treatment (**Figure 6B**) and a two-log decrease in total aerobic plate counts (day 0, treated vs. untreated) demonstrated the bactericidal effect of the treatment (**Figure 6C**). Analyses based on16s rRNA sequencing showed suggested that the pH treatment was effective at eliminating *V. chlorellavorus*, i.e., *V. chlorellavorus* reads were not detected in the pH-treated reactor 2 days after treatment but *V. chlorellavorus* comprised 25% of the bacterial community in the untreated reactor (**Figure 6D**).

**FIGURE 7:**
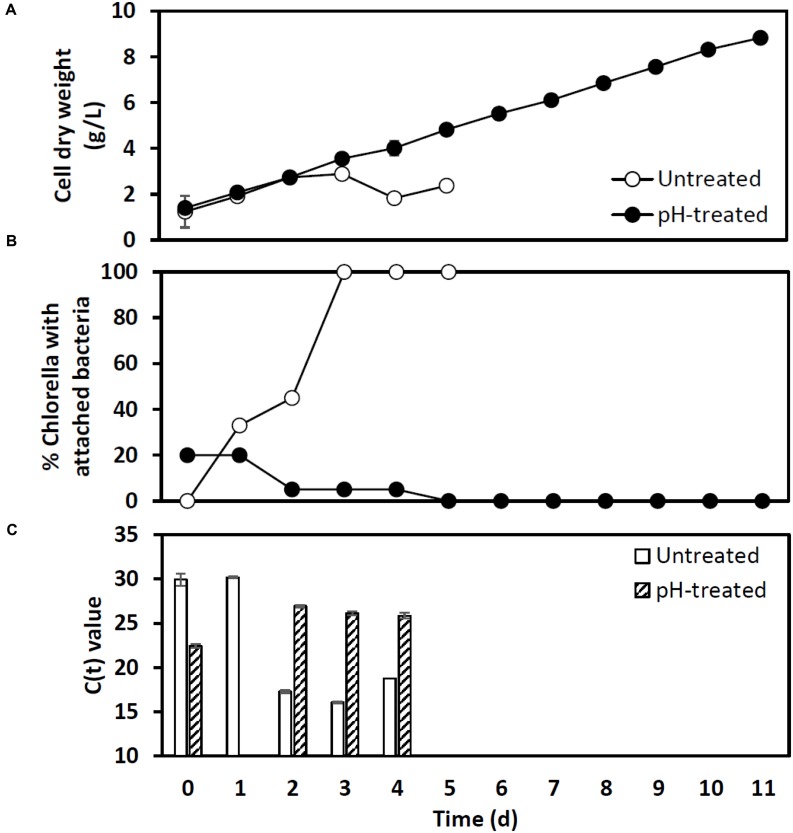
**Impact of the pH-shock treatment on (A) *Chlorella* cell dry weight, (B) bacterial attachment, and (C) qPCR assay *C*_t_ values for *Vampirovibrio chlorellavorous* from outdoor pilot scale reactors (1000 L, *n* = 1) inoculated with a contaminated *Chlorella* culture.** The *C*_t_ value, or cycle threshold, in **(C)** decreases as the target abundance increases. Lysates prepared for qPCR on day 1 from the pH treated reactor failed to amplify.

A second outdoor pH treatment experiment used the more sensitive and timely qPCR analysis to track infection (**Figure 7**). A mildly contaminated culture (20% bacterial attachment) removed from an open pond raceway (1000 L) was used to inoculate two additional 1000 L raceways that were operating mixotrophically. One raceway was left untreated while the other received a pH-shock treatment. Bacterial attachment in the untreated reactor dramatically increased to 100% within 3 days and cell dry weight declined and crashed within 3–5 days (**Figures 7A,B**). These changes followed an increase in detection of *V. chlorellavorus* in the untreated reactor via qPCR on day 2 when attachment was at 40% (**Figure 7B**). If this had been a single commercial scale reactor run, qPCR would have provided a 3-day warning that the culture was headed for a crash and crop protection strategies could have been initiated. *V. chlorellavorus* detection in the pH-treated culture remained stable, while attachment was reduced to 5% and cell dry weights continued to increase for 11 days to 8.5 g/L (**Figure 7**). This response confirmed the efficacy of the pH treatment against this previously fatal *V. chlorellavorus* predator in outdoor ponds used to grow *Chlorella.*

**FIGURE 8:**
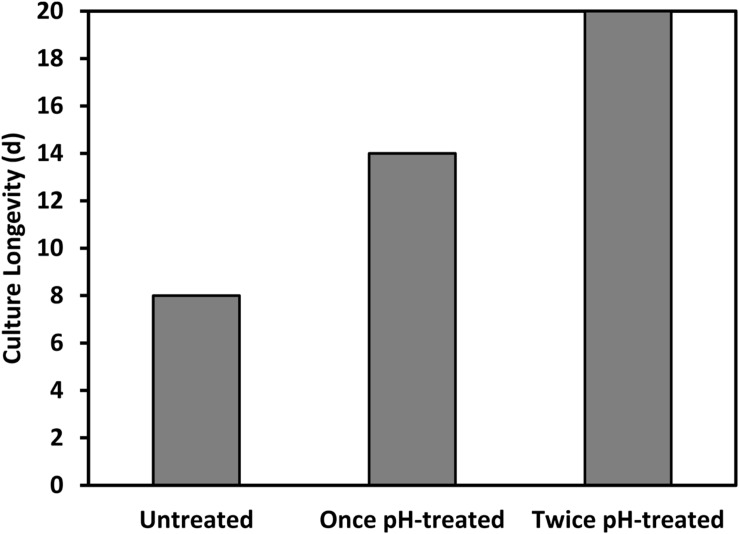
Longevity of a *Chlorella* culture was prolonged by applying the pH treatment repeatedly to the same culture upon transfer, at the beginning of at least two consecutive batch cycles.

To date, we have confirmed the efficacy of the pH-shock treatment at commercial-scale (130,000 L running volume) during nine additional reactor runs that were compared to untreated control cultures. The longevity of the pH treated ponds (12.7 ± 2.7 days [mean ± 1 SD]) was significantly higher (*t*-test; *t*(1) = 4.47, *P* < 0.01) than the longevity of the untreated ponds (7.0 ± 2.7 days [mean ± 1 SD]), increasing the total harvested biomass. Additionally, we found that for a typical batch operation involving scaling up and transferring cultures every 6 days between outdoor reactors, *Chlorella* could be treated right before transfer for at least two sequential transfers; this treatment schedule resulted in an increased culture longevity from eight to over twenty consecutive days (**Figure 8**).

## Discussion

Microalgae are among the few species in industrial microbiology that are grown in open ponds. Consequently, as in agriculture, crop protection is one of the most important challenges facing the microalgal industry, and it has been recognized as a chief limiting factor for microalgal production at commercial scale (Carney and Lane, 2014; McBride et al., 2016). Previously, pests encountered in commercial microalgal systems were not widely reported, and even minimized in an effort to make the microalgal industry appear more promising. Today, a diverse assemblage of zooplankton, fungi, bacteria, and viruses are known to attack microalgal cultures, and their impacts range from chronically reduced production to swift and irreversible culture crashes (Carney et al., 2014; Gong et al., 2015; Touloupakis et al., 2015; White and Ryan, 2015). Despite a more open acknowledgment of pests and the more frequent use of molecular diagnostic techniques for predator and pathogen detection (Carney et al., 2016), there are still very few detailed descriptions of crop protection strategies. Reports of treatments have included commercial fungicides (McBride et al., 2014) and hydrogen peroxide (Carney and Sorensen, 2015, U.S. Patent No 9,113,607) for fungal pathogens, pH transitions for rotifers (Zmora and Richmond, 2004), hyperchlorite (Zmora and Richmond, 2004), and size selective pulsed electric fields (Rego et al., 2015) for protozoa; see McBride et al. (2016) for a detailed review of reactive and preventative strategies.

Our study identified the causative organism that was apparently impacting our commercial *Chlorella* ponds. During infection, a larger proportion of *V. chlorellavorous* sequence reads were present in the region immediately surrounding the algal cells (i.e., the phycosphere) compared to bacteria free-living in the supernatant (**Figure 2**). This observation is in agreement with reports describing the epibiotic lifestyle of this predator (Coder and Star, 1978). Although *V. chlorellavorous* was originally identified as the potential causative crash agent via 16s rRNA sequencing (**Figures 1** and **2**), qPCR was relied upon for advanced warning of a culture crash via increasing *V. chlorellavorous* abundance (**Figure 7C**). In general, detection of *V. chlorellavorous* via qPCR preceded visual observations of an impending crash, including an increase in the percent of *Chlorella* cells with attached bacteria (> 20%; **Figures 6B** and **7B**), algal cell clumping and the culture changing color from dark green to yellowish brown (**Figure 5**). The analytical techniques developed and utilized here (i.e., *V. chlorellavorus*-specific qPCR assay, % bacterial attachment to *Chlorella* cells) proved important for determining timing and success of treatment applications.

Although largely unaccounted for in the literature, *V. chlorellavorus* is known as a devastating predator of *Chlorella*. We have experienced first-hand how this cyanobacterium can induce culture crashes in a matter of days. Likewise, other *Chlorella* ponds in the southwestern USA have suffered similar crises from this predator (personal communication from Drs. P. Lammers, J. Brown, and M. Sommerfeld). Molecular reports available in the NCBI GenBank database suggest that this predator is widespread globally and therefore could potentially affect *Chlorella* cultures regardless of their location. In this context, the development of a treatment against *V. chlorellavorus* is especially significant because (1) there was no treatment available prior and (2) *Chlorella*-like microalgae are among the most commonly produced crops by the industry. In addition, the treatment was opportunistically validated in outdoor mixotrophic cultures, which are one order of magnitude more productive than traditional photoautotrophic cultures (Ganuza et al., 2015; Ganuza and Tonkovich, 2016, U.S. Patent No 2015/0118735 A1).

**FIGURE 9:**
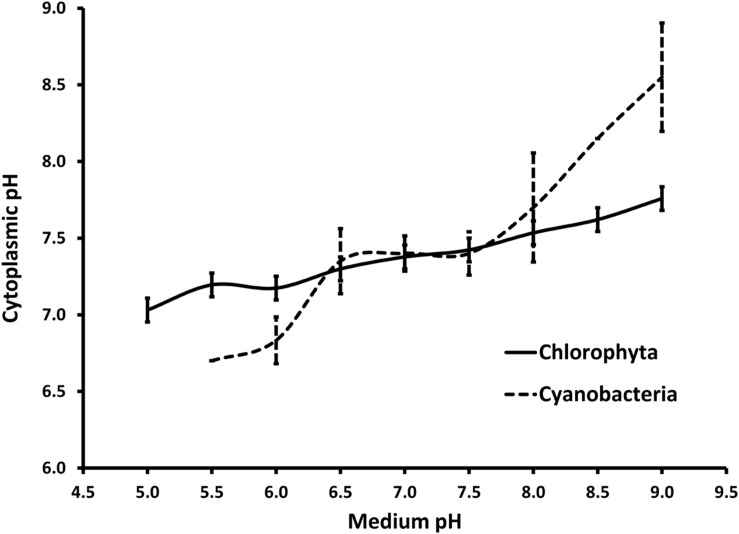
**Summary of cytoplasmic pH regulation in response to the pH in the surrounding media for members of phylum Chlorophyta and Cyanobacteria reported in the literature (seven and four members, respectively).** Chlorophyta includes *Chlorella kessleri* (El-Ansari and Colman, 2015), *Chlorella pyrenoidosa* and *Scenedesmus quadricauda* (Lane and Burris, 1981), *Chlorella saccharophila* (Gehl and Colman, 1985), *Chlorella vulgaris* and *Chlorella fusca* (Küsel et al., 1990) and *Dunaliella parva* (Gimmler et al., 1988). Cyanobacteria include *Agmenellum quadruplicatum* and *Gloeobacter violaceus* (Belkin et al., 1987), *Anacystis nidulans*, (Falkner and Horner, 1976) and *Synechococcus* sp. (Kallas and Castenholz, 1982). Note that only one Cyanobacterium (*A. quadruplicatrum*) did grow at a pH below 6.0. All other data points are expressed as mean values with error bars representing 1 standard deviation.

The crop protection strategy demonstrated here is straightforward and can be inexpensively applied at commercial scale (∼USD $100 for a 130,000 L reactor). The treatment has a low risk of failure given the broad tolerance of *Chlorella* to low pH. The pH-shock is highly selective against prokaryotes, as illustrated by the two log decrease in the total aerobic counts. Indeed, green microalgae (Chlorophyta) are better prepared than cyanobacteria to regulate internal pH relative to low external pH (relevant studies are summarized in **Figure 9**). Previously, the use of pH-shock treatment has been used to control lactic acid bacterial contamination from anaerobic yeast cultures in the Brazilian bioethanol industry (Basso et al., 2011). In microalgae, the transition to moderately low pH (6) has been used to control diatoms (Zmora and Richmond, 2004), while the transition to high pH (9–10) has been used to promote the growth of cyanobacteria over green microalgae (Vonshak et al., 1983; Touloupakis et al., 2015). Because the pH-shock treatment against *V. chlorellavorus* is applied for a discrete amount of time (15 min), resistance is less likely to develop than if applied continuously to the culture. To date, there is no indication that *V. chlorellavorus* has developed a tolerance to the treatment in our growth systems.

Successful rescue of actively infected algae culture heading for a crash is typically rare and attempts to circumvent a crash generally end in total loss when the culture is discarded. Total culture losses due to infections can greatly impact productivity, not to mention provide ample pathways to infect other ponds in the area during operation and take-down (reviewed by Carney and Lane, 2014; White and Ryan, 2015). The pH-shock treatment we have described here is capable of completely reversing high infection rates (>70% attachment) of a predatory bacterium, doubling *Chlorella* culture longevity and increasing the total harvested biomass from a commercial scale production platform. This treatment is now a routine component of our company mixotrophic operation.

## Author Contributions

EG and EL studied and reviewed the pH regulation in microalgae. EG and CS planned, designed, acquired, analyzed, and interpreted the data leading to the pH treatment development. LC and BB developed molecular assays and analyzed and interpreted the molecular data leading to the identification and monitoring of *V. chlorellavorus*. All authors drafted, read, critically revised and approved the final manuscript.

## Conflict of Interest Statement

The authors declare that the research was conducted in the absence of any commercial or financial relationships that could be construed as a potential conflict of interest. Heliae holds patents relevant to this work, all of which have been cited in this article.

## Footnotes

1 http://ftp.ncbi.nlm.nih.gov/pub/UniVec/

## References

[B1] Altschul S. F. Gish W. Miller W. Myers E. W. Lipman D. J. (1990). Basic local alignment search tool. *J. Mol. Biol.* 215 403–410. 10.1016/S0022-2836(05)80360-2 2231712

[B2] Basso L. Basso T. Rocha S. (2011). “Ethanol production in Brazil: The industrial process and its impact on yeast fermentation,” in *Biofuel Production-Recent Developments and Pospects*, ed. Bernardes M. A. S. (Rijeka: InTech Open Access Publisher), 85–100.

[B3] Bates L. Maechler M. Bolker B. Walker S. (2015). Fitting linear mixed-effects models using lme4. *J. Stat. Softw.* 67 1–48. 10.18637/jss.v067.i01

[B4] Beijerinck M. (1890). Kulturversuche mit Zoochlorellen, Lichenengonidien und anderen niederen Algen. *Bot. Ztg.* 48 729.

[B5] Belkin S. Mehlhorn R. J. Packer L. (1987). Proton gradients in intact cyanobacteria. *Plant Physiol.* 84 25–30. 10.1104/pp.84.1.25 11539679 PMC1056521

[B6] Carney L. T. Lane T. W. (2014). Parasites in algae mass culture. *Front. Microbiol.* 5:278 10.3389/fmicb.2014.00278 PMC4047527 24936200

[B7] Carney L. T. McBride R. C. Smith V. H. Lane T. W. (2016). “Molecular diagnostic solutions in algal cultivation systems,” in *Micro-Algal Production for Biomass and High-Value Products*, eds Slocombe S. P. Benemann J. R. (Boca Raton, FL: CRC Press).

[B8] Carney L. T. Reinsch S. S. Lane P. D. Solberg O. D. Jansen L. S. Williams K. P. (2014). Microbiome analysis of a microalgal mass culture growing in municipal wastewater in a prototype OMEGA photobioreactor. *Algal Res.* 4 52–61. 10.1016/j.algal.2013.11.006

[B9] Carney L. T. Sorensen K. (2015). *Methods for Treating a Culture of Haematococcus pluvialis for Contamination Using Hydrogen Peroxide. U.S. Patent No 9113607.* Washington, DC: U.S. Patent and Trademark Office.

[B10] Chelius M. K. Triplett E. W. (2001). The diversity of archaea and bacteria in association with the roots of *Zea mays* L. *Microb. Ecol.* 41 252–263. 10.1007/s002480000087 11391463

[B11] Coder D. Goff L. (1986). The host range of the chlorellavorus bacterium (“*Vampirovibrio chlorellavorus*”). *J. Phycol.* 46 543–547. 10.1111/j.1529-8817.1986.tb02499.x

[B12] Coder D. M. Starr M. P. (1978). Antagonistic association of the chlorellavorus bacterium (“*Bdellovibrio*” chlorellavorus) with *Chlorella vulgaris*. *Curr. Microbiol.* 1 59–64. 10.1007/BF02601710

[B13] Das K. C. Paul S. S. Sahoo L. Baruah K. K. Subudhi P. K. Ltu K. (2014). Bacterial diversity in the rumen of mithun (*Bos frontalis*) fed on mixed tree leaves and rice straw based diet. *Afr. J Microbiol. Res.* 8 1426–1433. 10.5897/AJMR2013.6507

[B14] El-Ansari O. Colman B. (2015). Inorganic carbon acquisition in the acid-tolerant alga *Chlorella kessleri*. *Physiol. Plant.* 153 175–182. 10.1111/ppl.12228 24828745

[B15] Falkner G. Horner F. (1976). pH Changes in the Cytoplasm of the Blue-Green Alga *Anacystis nidulans* Caused by Light-dependent Proton Flux into the Thylakoid Space. *Plant Physiol.* 58 717–718. 10.1104/pp.58.6.717 16659751 PMC542293

[B16] Ganuza E. Tonkovich A. L. (2015). *Method of Treating Bacterial Contamination in a Microalgae Culture with pH shock. U.S. Patent No 9181523.* Washington, DC: U.S. Patent and Trademark Office.

[B17] Ganuza E. Tonkovich A. L. (2016). “Heliae Development, LLC: an industrial approach to mixotrophy in microalgae,” in *Industrial biorenewables: a practical viewpoint*, ed. Dominguez de Maria P. (New York, NY: Wiley), 616.

[B18] Ganuza E. Tonkovich A. L. Licamele J. D. (2015). *Method of Culturing Microorganisms in Non-Axenic Mixotrophic Conditions. 41. U.S. Patent No 2015/0118735 A1.* Washington, DC: U.S. Patent and Trademark Office.

[B19] Gehl K. A. Colman B. (1985). Effect of external pH on the internal pH of *Chlorella saccharophila*. *Plant Physiol.* 77 917–921. 10.1104/pp.77.4.917 16664162 PMC1064631

[B20] Germond A. Hata H. Fujikawa Y. Nakajima T. (2013). The phylogenetic position and phenotypic changes of a chlorella-like alga during 5-year microcosm culture. *Eur. J. Phycol.* 48 485–496. 10.1080/09670262.2013.860482

[B21] Gimmler H. Kugel H. Leibfritz D. Mayer A. (1988). Cytoplasmic pH of *Dunaliella parva* and *Dunaliella acidophila* as monitored by in vivo ^31^P-NMR spectroscopy and the DMO method. *Physiol. Plant.* 74 521–530. 10.1111/j.1399-3054.1988.tb02013.x

[B22] Gong Y. Patterson D. J. Li Y. Hu Z. Sommerfeld M. Chen Y. (2015). *Vernalophrys algivore* gen. nov., sp. nov. (Rhizaria: Cercozoa: Vampyrellida), a new algal predator isolated from outdoor mass culture of *Scenedesmus dimorphus*. *Appl. Environ. Microbiol.* 81 3900–3913. 10.1128/AEM.00160-15 25819973 PMC4524137

[B23] Gromov B. V. Mamkaeva K. A. (1972). Electron microscopic study of parasitism by *Bdellovibrio chlorellavorus* bacteria on cells of the green alga *Chlorella vulgaris*. *Tsitologiia* 14 256–260. 5011884

[B24] Guerrero R. Pedros-Alio C. Esteve I. Mas J. Chase D. Margulis L. (1986). Predatory prokaryotes: predation and primary consumption evolved in bacteria. *Proc. Natl. Acad. Sci. U.S.A.* 83 2138–2142. 10.1073/pnas.83.7.2138 11542073 PMC323246

[B25] Holmberg A. Blomstergren A. Nord O. Lukacs M. Lundeberg J. Uhlén M. (2005). The biotin-Streptavidin interaction can be reversibly broken using water at elevated temperatures. *Electrophoresis* 26 501–510. 10.1002/elps.200410070 15690449

[B26] Hoshina R. Fujiwara Y. (2013). Molecular characterization of chlorella cultures of the national institute for environmental studies culture collection with description of *Micractinium inermum* sp. nov., *Didymogenes sphaerica* sp. nov., and *Didymogenes soliella* sp. nov. (Chlorellaceae, Trebouxiphyceae). *Phycol. Res.* 61 124–132.

[B27] Kallas T. Castenholz R. W. (1982). Rapid transient growth at low pH in the cyanobacterium *Synechococcus* sp. *J. Bacteriol.* 149 237–246. 6798020 10.1128/jb.149.1.237-246.1982 PMC216615

[B28] Küsel A. C. Sianoudis J. Leibfritz D. Grimme L. H. Mayer A. (1990). The dependence of the cytoplasmic pH in aerobic and anaerobic cells of the green algae *Chlorella fusca* and *Chlorella vulgaris* on the pH of the medium as determined by ^31^P in vivo NMR spectroscopy. *Arch. Microbiol.* 153 254–258. 10.1007/BF00249077

[B29] Lane A. E. Burris J. E. (1981). Effects of environmental pH on the internal pH of *Chlorella pyrenoidosa*, *Scenedesmus quadricauda*, and *Euglena mutabilis*. *Plant Physiol.* 68 439–442. 10.1104/pp.68.2.439 16661932 PMC427506

[B30] Mamkaeva K. A. Rybal’chenko O. V. (1979). Ultrastructural characteristics of *Bdellovibrio chlorellavorus*. *Mikrobiologiia* 48 159–161. 423805

[B31] Marin B. (2012). Nested in the chlorellales or independent class? Phylogeny and classification of the Pedinophyceae (Viridiplantae) revealed by molecular phylogenetic analyses of complete nuclear and plastid-encoded rRNA operons. *Protist* 163 778–805. 10.1016/j.protis.2011.11.004 22192529

[B32] Marin B. Palm A. Klingberg M. Melkonian M. (2003). Phylogeny and taxonomic revision of plastid-containing euglenophytes based on SSU rDNA sequence comparisons and synapomorphic signatures in the SSU rRNA secondary structure. *Protist.* 154 99–145. 10.1078/143446103764928521 12812373

[B33] McBride R. C. Lopez S. Meenach C. Burnett M. Lee P. A. Nohilly F. (2014). Contamination management in low cost open algae ponds for biofuels production. *Ind. Biotechnol.* 10 221–227. 10.1089/ind.2013.1614

[B34] McBride R. C. Smith V. H. Carney L. T. Lane W. T. (2016). “Crop protection in open ponds,” in *Micro-Algal Production for Biomass and High-Value Products*, eds Slocombe S. P. Benemann J. R. (Boca Raton, FL: CRC Press).

[B35] McMurdie P. J. Holmes S. (2013). Phyloseq: an R package for reproducible interactive analysis and graphics of microbiome census data. *PLoS ONE* 8:e61217 10.1371/journal.pone.0061217 PMC3632530 23630581

[B36] Pruesse E. Peplies J. Glöckner F. O. (2012). SINA: accurate high-throughput multiple sequence alignment of ribosomal RNA genes. *Bioinformatics* 28 1823–1829. 10.1093/bioinformatics/bts252 22556368 PMC3389763

[B48] R Core Team (2016). *R: Language and Enviroment for Statistical Computing*. Vienna: R Foundation for Statistical Computing Available at: http://www.R-project.org/

[B37] Rego D. Redondo L. M. Geraldes V. Costa L. Navalho J. Pereira M. T. (2015). Control of predators in industrial scale microalgae cultures with Pulsed Electric Fields. *Bioelectrochemistry* 103 60–64. 10.1016/j.bioelechem.2014.08.004 25220563

[B38] Safi C. Zebib B. Merah O. Pontalier P.-Y. Vaca-Garcia C. (2014). Morphology, composition, production, processing and applications of *Chlorella vulgaris*: a review. *Renew. Sustain. Energy Rev.* 35 265–278. 10.1016/j.rser.2014.04.007

[B39] Shi C. Wang C. Xu X. Huang B. Wu L. Yang D. (2015). Comparison of bacterial communities in soil between nematode-infected and nematode-uninfected *Pinus massoniana* pinewood forest. *Appl. Soil Ecol.* 85 11–20. 10.1016/j.apsoil.2014.08.008

[B40] Soo R. M. Skennerton C. T. Sekiguchi Y. Imelfort M. Paech S. J. Dennis P. G. (2014). An expanded genomic representation of the phylum Cyanobacteria. *Genome Biol. Evol.* 6 1031–1045. 10.1093/gbe/evu073 24709563 PMC4040986

[B41] Soo R. M. Woodcroft B. J. Parks D. H. Tyson G. W. Hugenholtz P. (2015). Back from the dead; the curious tale of the predatory cyanobacterium *Vampirovibrio chlorellavorus*. *PeerJ.* 3:e968 10.7717/peerj.968 PMC4451040 26038723

[B42] Tonkovich A. L. Ganuza E. Licamele J. D. Galvez A. Sullivan T. J. Adame T. (2014). *Large Scale Mixotrophic Systems. WO. Patent No 2014/144270 A1.* Geneva: World Intellectual property Organization.

[B43] Touloupakis E. Cicchi B. Benavides A. M. S. Torzillo G. (2015). Effect of high pH on growth of *Synechocystis* sp. *PCC* 6803 cultures and their contamination by golden algae (*Poterioochromonas* sp.). *Appl. Microbiol. Biotechnol.* 100 1333–1341. 26541331 10.1007/s00253-015-7024-0 PMC4717179

[B44] Vonshak A. Boussiba S. Abeliovich A. Richmond A. (1983). Production of Spirulina biomass: maintenance of monoalgal culture outdoors. *Biotechnol. Bioeng.* 25 341–349. 18548655 10.1002/bit.260250204

[B45] White R. L. Ryan R. A. (2015). Long-term cultivation of algae in open-raceway ponds: messons from the field. *Ind. Biotechol.* 11 213–220. 10.1089/ind.2015.0006

[B46] Wong A. L. C. Beebee T. J. C. Griffiths R. A. (1994). Factors affecting the distribution and abundance of an unpigmented heterotrophic alga *Prototheca richardsi*. *Freshw. Biol.* 32 33–38. 10.1111/j.1365-2427.1994.tb00863.x

[B47] Zmora O. Richmond A. (2004). “Microalgae for aquaculture: microalgae production for aquaculture,” in *Handbook of Microalgal Culture*, ed. Richmond A. (Oxford: Blackwell Publishing Ltd), 365–379.

